# *C. elegans* PAT-9 is a nuclear zinc finger protein critical for the assembly of muscle attachments

**DOI:** 10.1186/2045-3701-2-18

**Published:** 2012-05-22

**Authors:** Qian Liu, Rebecca A Bachmann, Mitchell Meghpara, Lauren Rogowski, Benjamin D Williams

**Affiliations:** 1 Department of Cell and Developmental Biology, University of Illinois at Urbana-Champaign, 601 S. Goodwin Ave, B107 Chemical and Life Sciences Laboratory, Urbana, IL, 61801, USA; 2 Present Address: Boston Biomedical Research Institute, 64 Grove St., Watertown, MA, 02472, USA

**Keywords:** Sarcomere, Muscle, Zinc finger, Pat

## Abstract

**Background:**

*Caenorhabditis elegans* sarcomeres have been studied extensively utilizing both forward and reverse genetic techniques to provide insight into muscle development and the mechanisms behind muscle contraction. A previous genetic screen investigating early muscle development produced 13 independent mutant genes exhibiting a Pat (paralyzed and arrested elongation at the two-fold length of embryonic development) muscle phenotype. This study reports the identification and characterization of one of those genes, *pat-9*.

**Results:**

Positional cloning, reverse genetics, and plasmid rescue experiments were used to identify the predicted *C. elegans* gene T27B1.2 (recently named *ztf-19*) as the *pat-9* gene. Analysis of *pat-9* showed it is expressed early in development and within body wall muscle lineages, consistent with a role in muscle development and producing a Pat phenotype. However, unlike most of the other known Pat gene family members, which encode structural components of muscle attachment sites, PAT-9 is an exclusively nuclear protein. Analysis of the predicted PAT-9 amino acid sequence identified one putative nuclear localization domain and three C2H2 zinc finger domains. Both immunocytochemistry and PAT-9::GFP fusion expression confirm that PAT-9 is primarily a nuclear protein and chromatin immunoprecipitation (ChIP) experiments showed that PAT-9 is present on certain gene promoters.

**Conclusions:**

We have shown that the T27B1.2 gene is *pat-9*. Considering the Pat-9 mutant phenotype shows severely disrupted muscle attachment sites despite PAT-9 being a nuclear zinc finger protein and not a structural component of muscle attachment sites, we propose that PAT-9 likely functions in the regulation of gene expression for some necessary structural or regulatory component(s) of the muscle attachment sites.

## Introduction

The nematode *C. elegans* provides an established, developmentally well-documented, and evolutionarily conserved system to study muscle structure, development, and function [1,2]. The *C. elegans* sarcomere, the basic muscle contraction unit, has been studied for decades revealing a highly organized structure consisting of several hundred proteins, yet new components are still being identified [2-6]. In *C. elegans* sarcomeres*,* myosin thick filaments are organized around M-lines and actin thin filaments are anchored to the dense bodies, structures analogous to the vertebrate Z-disk. The dense bodies and M-lines are sites of attachment for body wall muscle cells to the basement membrane, thus transmitting the force of muscle contraction and allowing movement [7]. The overall mechanism of *C. elegans* muscle function is highly evolutionarily conserved and many of the known proteins have vertebrate orthologs within vertebrate muscle costameres or non-muscle focal adhesions [1,2,6,8].

Many of the components necessary for *C. elegans* muscle attachments were identified by immunological approaches or through genetic screening for mutants exhibiting disorganized myofilaments, paralysis, and/or embryonic arrest [4,9,10]. Genes required for muscle development and function are grouped into two main phenotypic classes of mutants, Pat (paralyzed and arrested elongation at the two-fold length of embryonic development) and Unc (Uncoordinated), with some genes capable of producing both phenotypes depending on the nature of the mutation [10,11]. Both of these phenotypic classes comprise proteins that localize specifically to the M-lines, dense bodies or both, and much of their organized assembly into functional sarcomeres has been characterized (reviewed in [1,7]). A fourth type of localization for some sarcomeric proteins is exemplified by ZYX-1, UNC-95, UNC-97, and UNC-98, which are found both at the sarcomere and in the nucleus, supporting an additional role for the sarcomere as a platform for mediating signal transduction to the nucleus to influence gene expression [12-15].

In a continuing effort to identify new components required for muscle attachment site assembly, we have focused on characterizing the Pat group of *C. elegans* mutants. Mutations in the genes that encode the membrane-associated components of the muscle attachment sites, such as *unc-52, pat-2, pat-3, unc-112, unc-97, pat-4 and pat-6*, display the most severe Pat phenotype, in which neither thin filaments nor thick filaments are assembled into the myofilament lattice [10]. Previous studies indicate that PAT-4, UNC-97 and PAT-6 serve as intermediary members of the linkage formed between the integrins and myofilaments [16-18]. Here, we identified and characterized a novel gene in the Pat family, *pat-9*[10,19]. In *pat-9* mutants, the recruitment of both actin thin filaments and myosin thick filaments to the muscle cell membrane appears to be disrupted. Similar to other Pat genes, *pat-9* is expressed in *C. elegans* body wall muscle during embryogenesis. Unlike other genes in the Pat family that are structural and functional components of muscle attachments, *pat-9* encodes a nuclear localized C2H2 zinc finger transcription factor, suggesting a potential regulatory role, as opposed to a sarcomeric structural function for PAT-9.

## Results

### The *pat-9* gene is encoded by T27B1.2 (*ztf-19*)

**Figure 1:**
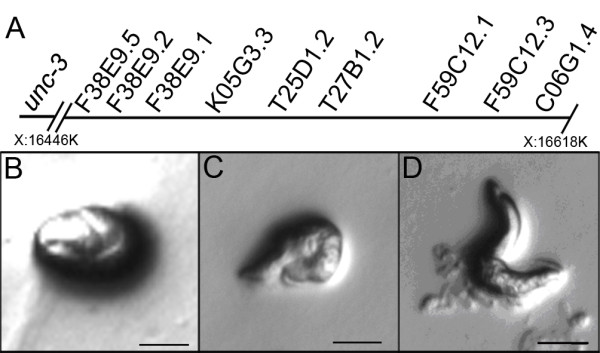
**RNAi for T27B1.2 produces a Pat phenotype.A**) Nine candidate genes located near the mapped *pat-9* locus on the X chromosome show muscle expression patterns according to SAGE data. **B**) Wild type N2 animals at the 2.5-fold stage **C**) *pat-9* mutant animals segregated from strain RW1385. **D**) Only T27b1.2 RNAi caused a Pat phenotype at the embryonic stage. Bars = 10 μm.

The pat-9 gene was genetically mapped to the right end of the X chromosome [19]. Subsequently, a combination of positional cloning and candidate gene approaches were used to identify the *pat-9* gene. Single nucleotide polymorphism (SNP) mapping data localized *pat-9(st558)* to the right of the F38E9 marker on chromosome X, near the T25D1 marker, since no recombination events were detected between this marker and *pat-9* (Additional file 1: Table S1). There are 23 genes in the 200 kb region centered at T25D1, of which 9 are expressed in muscle based on SAGE tag data (Figure 1A and Additional file 1: Table S2) from the WormBase website (http://www.wormbase.org, Release WS223). Therefore, RNA interference (RNAi) was performed on each of the nine candidate genes. The RNAi for T27B1.2 was the only one that produced a significant number (10%, N > 200) of phenotypic Pat-like F1 progenies (Figure 1D compared with 1B, N2 and 1 C *pat-9*). Performing RNAi for the other eight candidate genes resulted in normal embryos. Our data was recently corroborated by Meissner et al. who conducted an RNAi screen for new Pat genes and identified T27B1.2 as having a Pat loss of function phenotype [20]. This data strongly implicates T27B1.2 as *pat-9*.

**Figure 2:**
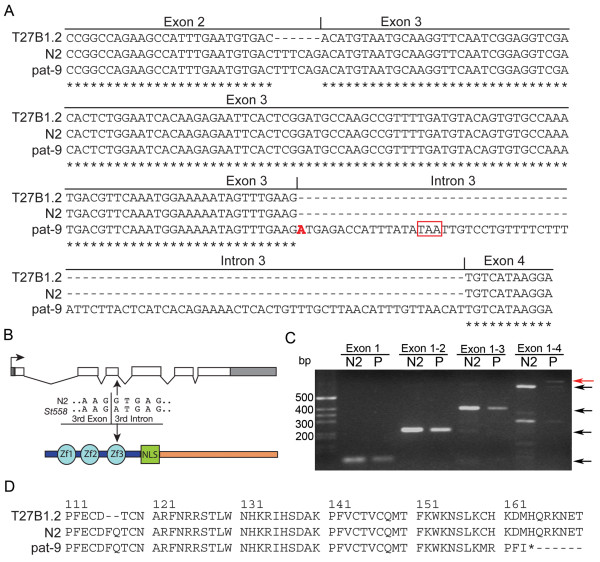
**Verification that T27B1.2 is*pat-9.*A**) The sequence of T27B1.2 amplified from *pat-9* mutant animals shows a G→A transversion mutation in the splicing donor site of the third exon/intron junction. DNA sequence alignment of the amplified pat-9 cDNA from N2 and *pat-9* animals compared with the predicted T27b1.2 gene model from WormBase shows six additional nucleotides in exon 3 and predicts a truncated protein in *pat-9* animals. The single mutated base change in *pat-9* is highlighted in red and the in-frame stop codon is boxed in red. The remaining sequence alignments are identical (not shown). **B**) The point mutation at the splicing donor site predicts the subsequent incorporation of intron 3, disrupting the third Zn finger and generating a premature in-frame stop codon at amino acid 164. **C**) RT-PCR from RNA extracted from N2 and *pat-9* (P) worms was performed using oligonucleotide primers specific for exons 1, 2, 3, or 4 and shows that intron 3 is incorporated into the mRNA of *pat-9* animals (red arrow) but not N2 animals. Black arrows indicate properly spliced products. **D**) Analysis of the predicted PAT-9 amino acid sequence indicates that *pat-9* animals produce a truncated protein of 163 amino acids.

The hypothetical gene T27B1.2, identified by the *C. elegans* genome-sequencing consortium near the right end of chromosome X, is predicted to encode a C2H2 zinc finger protein and was recently placed into the zinc finger transcription factor family and renamed *ztf-19*; however, the gene will be referred to as T27B1.2. To confirm T27B1.2 is *pat-9*, a transformation rescue experiment was performed. Injection of a genomic DNA fragment containing T27B1.2 and 3.3 kb of upstream noncoding genomic DNA completely rescued the phenotype of *pat-9* homozygotes indicating the causal mutation resides in the T27B1.2 gene. To identify the *pat-9(st558)* mutation at the molecular level, the T27B1.2 gene was amplified and sequenced from *pat-9(st558)*, segregated by strain RW1385. DNA sequence analysis revealed a single point mutation (G→A) in the putative splice donor site of the 3 rd intron (Figure 2A and B). The G residue at this position is completely conserved (~100%) in *C. elegans* splice donor sites, and mutation of this G residue is predicted to lead to aberrant splicing resulting in a non-functional PAT-9 protein. However, since the splice junctions are predicted based on a gene model and had not yet been verified, total RNA was extracted from both *pat-9* mutant animals and N2 animals and subjected to reverse transcriptase PCR to amplify T27B1.2 cDNA using primers specific to each exon to test for intron incorporation into the mature *pat-9* mRNA (Figure 2C). As predicted by the gene sequence, the point mutation resulted in mis-splicing of exon 3 to exon 4 resulting in the inclusion of the third intron (Figure 2C). DNA sequencing of this mis-spliced product confirmed the inclusion of the intron. Based on the *pat-9* cDNA sequence, the predicted amino acid sequence is truncated due a premature stop codon in the mutant *pat-9* mRNA (Figure 2D). Also, sequencing identified an additional 6 nucleotides between predicted exon 2 and exon 3 (Figure 2A) indicating *pat-9* is spliced differently from the gene model displayed on WormBase (http://www.wormbase.org). Together, this evidence provides unambiguous confirmation that the identity of *pat-9* is T27B1.2.

### PAT-9 is localized to nuclei through all developmental stages

**Figure 3:**
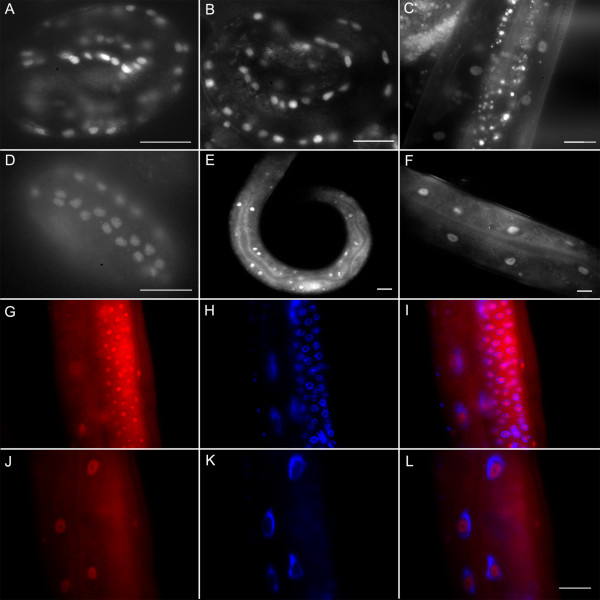
**Characterization of PAT-9 expression.** Localization of the PAT-9 protein as determined by use of a PAT-9::GFP translational fusion in the transgenic rescued *pat-9(st558)* stain. Fluorescence microscopy shows the localization of PAT-9::GFP within muscle nuclei in a **A**) 2-fold embryo, **B**) larva and **C**) adult animal. The anti-PAT-9 antibody stains body wall muscle nuclei of wild-type embryos (**D**), as well as the nuclei of young larvae (**E**) and adults (**F**); PAT-9 immunostaining shows that PAT-9 is expressed in germ-like nuclei in cytoplasmic syncytium of the *C. elegans* gonad (**G**). Endogenous PAT-9 (**G and J**) is nuclear and localized to the DAPI-poor region as shown by (**H and K**) DAPI staining, and (**I and L**) merging with PAT-9 immunostaining. Bars =10 μm.

To characterize the PAT-9 protein, PAT-9 expression was analyzed in embryos, larvae and adults using a functional *pat-9::gfp* transgene and confirmed by immunostaining for endogenous PAT-9 (Figure 3). Since the DNA used for rescuing the Pat-9 phenotype contained 3.3 kb of genomic sequence upstream of the translational start site, this region of DNA was considered to be sufficient to function as the *pat-9* promoter and was therefore used to generate a *pat-9* transgene expression plasmid. The transgene was designed to express full length PAT-9, with GFP fused to its amino terminus in the vector pPD118.20. Expression of PAT-9::GFP from this plasmid was first detected in the early embryo; by the 2-fold stage of development PAT-9::GFP localized exclusively to the body wall muscle nuclei in each body wall muscle quadrant (Figure 3A). PAT-9::GFP continued to be expressed in the cell nuclei of larvae and adults, and it became no longer exclusive to body wall muscle (Figure 3B and C). To confirm the transgenic expression pattern, antiserum against the PAT-9 carboxyl terminus, which does not contain the predicted zinc fingers, was generated (Additional file 1: Figure S1). The PAT-9 immunostaining of N2 animals showed a similar expression pattern to that seen with the *pat-9* transgene. Endogenous PAT-9 was expressed from very early stages and throughout all developmental stages (Figure 3D, E and F), and in adults endogenous PAT-9 was predominantly nuclear and appeared excluded from the DAPI-poor nucleoli (Figure 3G-L). In addition to expression in body wall muscle, PAT-9 was observed in the germ cell-like nuclei of the cytoplasmic syncytium of the *C. elegans* gonad, where most nuclei are arrested in meiosis (Figure 3G, H and I). Overall, the expression profile for PAT-9 is consistent with its involvement in muscle development; however, its apparent subcellular localization indicates that it is not a stable structural component of the sarcomere.

### PAT-9 has a nuclear localization signal (NLS)

**Figure 4:**
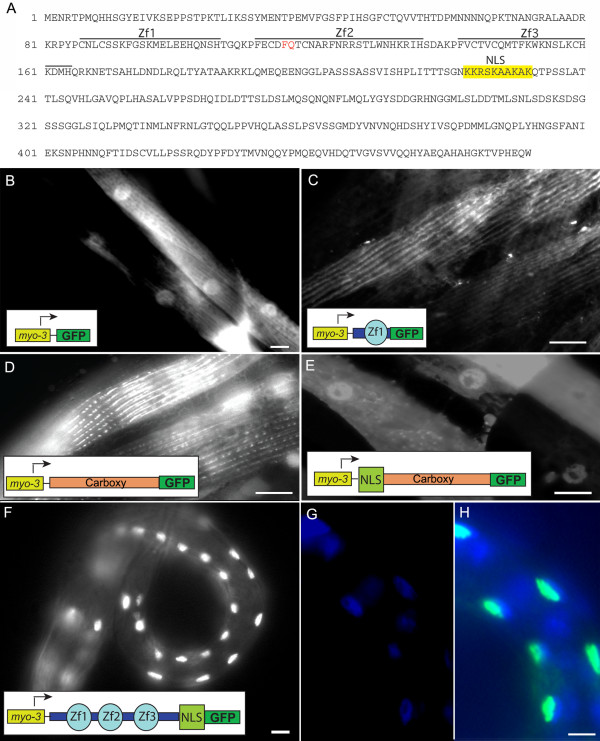
**PAT-9 contains a lysine/arginine-rich NLS.A**) The amino acid sequence predicted from the pat-9 cDNA cloned from N2 worms is shown. Each of the three putative zinc fingers is underlined. The additional two amino acids not present in the WormBase model are shown in red. The deduced NLS is highlighted in yellow. B-H) Plasmids expressing truncations of PAT-9 fused to GFP under control of the *myo-3* promoter were used to identify the PAT-9 NLS. GFP expression from transgenic animals carrying extrachromosomal arrays of **B**) *P*_*myo-3*_*::gfp* (pPD118.20) expressing GFP alone, **C**) *P*_*myo-3*_*::pat-9zf1::gfp*, containing the amino terminal sequence and the first zinc finger of PAT-9, **D**) *P*_*myo-3*_*::pat-9 C::gfp* containing only the carboxyl terminal sequence, **E**) *P*_*myo-3*_*::pat-9CNLS::gfp* containing the carboxyl terminal sequence with the putative NLS, **F**) *P*_*myo-3*_*::pat-9zf123NLS::gfp* containing three zinc fingers and the predicted NLS. The exclusively nuclear localization of *P*_*myo-3*_*::pat-9zf123NLS::gfp* was confirmed by G) DAPI staining and H) merging of GFP and DAPI images. Bars = 10 μm.

Analysis of the PAT-9 sequence for putative conserved domains using the Pfam protein conserved domain database (http://www.pfam.org) identified 3 putative C2H2 zinc fingers (aa 86–106, aa 112–136, aa 142–164), but no predicted NLS despite the strong nuclear localization of PAT-9. However, further analysis identified one region near the middle of the protein (aa 223–232) that contained five lysines (K) and an arginine (R) (Figure 4A) spaced so as to adhere to two proposed optimal consensus sequence requirements [K(R/K)X(R/K)] and [KR(R/X)K] of a classical NLS [21] suggesting this could be the PAT-9 NLS. To experimentally identify the PAT-9 NLS, a series of truncations of *pat-9* were generated and fused to GFP for transgenic expression analysis. The *myo-3* promoter was used to specifically express all transgenes in body wall muscle. A transgenic line with *P*_*myo-3*_*::gfp* (pPD118.20) was used as a control; images of this show GFP localization throughout the muscle cell, including the nucleus, which is as expected since GFP enters the nucleus in the absence of an NLS due to its small size (Figure 4B). Transgenic animals expressing *P*_*myo-3*_*::zf1::gfp*—containing the amino terminal sequence and the first putative zinc finger, or *P*_*myo-3*_*::pat-9 C::gfp*—containing the carboxyl terminal half of PAT-9, localized exclusively to the cytoplasm suggesting that there was no NLS in these regions (Figure 4C and D). Interestingly, both of these truncated PAT-9::GFP fusions showed an apparent sarcomeric localization, which may suggest a transient sarcomeric localization for PAT-9 prior to shuttling into the nucleus similar to UNC-95, UNC-97, UNC-98, and ZXY-1. However, when the putative NLS was added to the carboxyl terminal half of PAT-9, the GFP fusion protein strongly localized to the nucleus (Figure 4, compare D with E). Since the GFP fusion proteins are too large to passively diffuse into the nucleus, the observed nuclear localization must have been achieved through active import. Furthermore, GFP fused to an amino terminal portion of PAT-9 that includes the same putative NLS localized exclusively to muscle nuclei (Figure 4F–H). Taken together, the ten amino acid stretch containing the hypothetical NLS does indeed function as the PAT-9 NLS.

### PAT-9 associates with gene promoters

**Figure 5:**
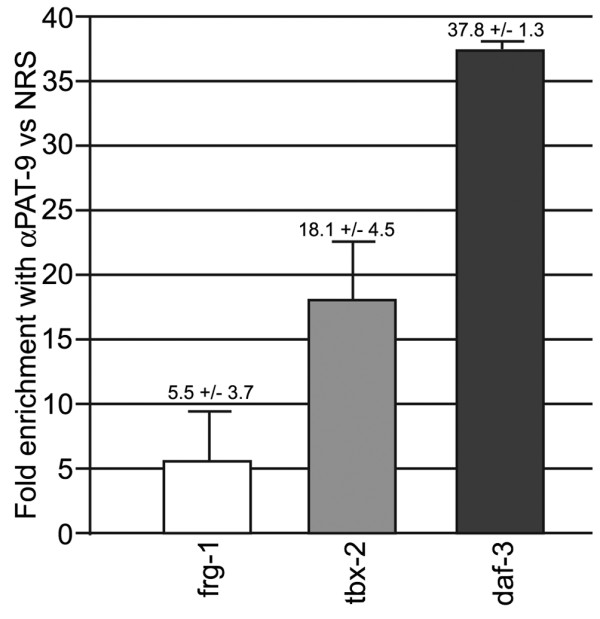
**PAT-9 is associated with gene promoters in vivo.** ChIP experiments show enrichment of the indicated 5′ promoter DNA with the αPAT-9 antibody compared with NRS control as assayed by qPCR. The *tbx-2* and *daf-3* bars represent the results from three independent ChIP experiments and the *frg-1* bar represents the results from six independent ChIP experiments. All qPCRs were performed as five replicates to generate one data point. Fold enrichment was calculated as 2e (Ct _NRS_ – Ct _PAT-9_).

The nuclear localization of PAT-9 along with the identification of 3 putative C2H2 zinc fingers in *pat-9* suggests that PAT-9 could be a nuclear C2H2 zinc finger transcription factor that localizes to promoters and regulates gene expression. A yeast one-hybrid screen aimed at identifying *C. elegans* regulatory sequences bound by transcription factors predicted five promoters that could be bound by PAT-9/ZTF-19 [22]. To verify these potential interactions in vivo, chromatin immunoprecipitation (ChIP) experiments were performed using the PAT-9 polyclonal antibody. Two of the candidate promoters, *daf-3* and *tbx-2*, were chosen for ChIP experiments as well as a control gene, *frg-1*, not known to bind PAT-9. Cultures of mixed stage N2 animals were collected, fixed with formaldehyde, and used for ChIP with either PAT-9 antibody or normal rabbit serum (NRS). The associated DNAs were extracted, purified, and subjected to quantitative real-time PCR (qPCR). The cross threshold (Ct) values (of which a smaller Ct number indicates a larger amount of the target DNA in the original sample) were determined for each promoter and the control, and the fold enrichment for each DNA in the PAT-9 ChIPs compared with the DNA in NRS ChIPs were calculated (Figure 5). Both the *daf-3* (37.8 fold) and *tbx-2* (18.1 fold) DNAs were strongly enriched in the PAT-9 ChIPs indicating that PAT-9 was highly bound to their promoters in vivo. Surprisingly, *frg-1* was slightly enriched (5.5 +/− 3.7 fold) in the PAT-9 ChIPs, despite not being a one-hybrid hit. Potentially accounting for this, *frg-1*, similar to *pat-9*, is expressed throughout development and within muscle, and may, in fact, be regulated by PAT-9 [6]. Overall, our results indicate that PAT-9 interacts with gene promoters in vivo and likely regulates the expression of additional genes not identified by the one-hybrid approach, at least one of which must account for the Pat-9 phenotype.

### PAT-9 is required for the assembly of functional muscle attachments

**Figure 6:**
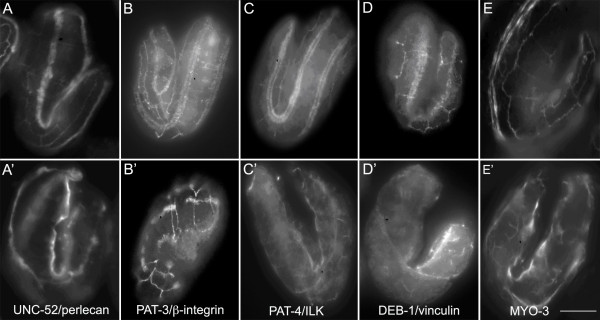
**Muscle attachment sites are disrupted in*pat-9*mutants.** N2 (A, B, C, D and E) and *pat-9(st558)* (A’, B’, C’ D’ and E’) embryos were stained with monoclonal antibodies to UNC-52/perlecan (**A and A’**), PAT-3/beta-integrin (**B and B’**), PAT-4/ILK (**C and C’**), DEB-1/vinculin (**D and D’**) and MYO-3/MHC-A (**E and E’**). All five proteins are expressed in *pat-9(st558)* embryos indicating PAT-9 does not regulate these genes. Arrows show the structures stained by each antibody. All the animals were co-stained with monoclonal antibody MH27 to visualize the hypodermal cell junctions, enabling the identification of the orientation and age of the embryos. Bar = 10 μm.3^rd^ exon.

In Pat mutants, including *pat-9*, actin and myosin filaments are not recruited properly to muscle attachment sites suggesting that the assembly of muscle attachments is disrupted. The PAT-9 nuclear localization and ChIP data suggests a gene regulatory role for PAT-9 in the expression of genes encoding muscle attachment structural components. Thus, the expression and localization of known muscle attachment proteins was investigated in *pat-9* mutants. The *pat-9*(*st558*) allele was used to ablate functional PAT-9 expression and the embryos were stained with antibodies to components of muscle attachments (Figure 6). Immunostaining showed that UNC-52/perlecan (Figure 6A’), PAT-3/integrin (Figure 6B’), PAT-4/ILK (Figure 6C’), DEB-1/vinculin (Figure 6D’), and myosin (Figure 6E’), were all expressed in *pat-9(st558)* embryos. In wild type embryos, PAT-3/integrin, PAT-4/ILK, and DEB-1/vinculin are located in ordered arrays of dense bodies and M lines (Figure 6A–E). In contrast, in *pat-9(st558)* mutant embryos, these proteins are not well organized and UNC-52/perlecan was not located in a continuous strip in the basal lamina of *pat-9(st558)* mutants. Muscle attachments have a distinct assembly pathway beginning with proteins such as UNC-52/perlecan [2]; therefore, when early components fail to properly assemble, then downstream components such as PAT-3/integrin, PAT-4/ILK, DEB-1/vinculin and myosin are not well organized either. In wild-type animals, myofilaments are organized, recognizable, and attached to the muscle cell membrane through muscle attachments forming two arrays of muscle cells that are parallel in each body wall muscle quadrant. In many *pat-9* mutant embryos, the dorsal body-wall muscle quadrants have pulled away from the body wall: direct evidence that the muscle attachments have not been properly formed. Furthermore, myosin filaments are not recruited properly in *pat-9(st558)* mutant embryos. Thus, although many components of muscle attachments are expressed in these mutants, they often fail to form functional muscle attachments and seem to require PAT-9 for their proper assembly.

## Discussion

We have shown that the *pat-9* gene is the predicted gene T27B1.2, renamed *ztf-19*, which encodes a nuclear C2H2 type zinc finger transcription factor. Thus, PAT-9 is the only exclusively nuclear protein among all *pat* genes that have been identified and characterized. In mammals, many DNA-binding transcriptional regulators contain C2H2 zinc fingers [23]. Similarly, in *C. elegans* C2H2 zinc finger transcription regulators play critical roles in many biological aspects, including body wall muscle development, synaptic transmission, and egg-laying behavior and fertility [15,24,25]. The characterization of PAT-9 suggests it functions as a transcription factor for one or more factors required in muscle attachment site assembly. Lending support to this hypothesis, a yeast one-hybrid binding assay showed that PAT-9/ZTF-19 bound to five promoters, *daf-3tbx-2cog-1let-7*, and *mir-76*[22]. Furthermore, two of the genes, *daf-3* and *tbx-2*, were shown to be bound by PAT-9 in vivo. Interestingly, both genes are transcriptional regulators themselves involved in *C. elegans* pharynx development, while *daf-3* is additionally involved in dauer formation, indicating that PAT-9 has multiple roles during development including roles outside of body wall muscle development [26-28].

TBX-2 is a transcription factor specifically required for development of ABa-derived pharyngeal muscles [26]. TBX-2 is expressed from early embryo to adult stages and *tbx-2::gfp* expression was also observed in body wall muscles at the comma stage and later, in 2- to 3-fold embryos similar to the expression of *pat-9*. As with *pat-9*, there is a muscle defect in *tbx-2* mutants at the embryo stage. Interestingly, RNAi analysis reveals that *tbx-2* is critical for early larval development and normal locomotion. These animals have a less severe phenotype than the *pat-9* RNAi animals that have embryo lethality and larval arrest, but are still consistent with TBX-2 being a downstream target of PAT-9 [29,30].

DAF-3 is a transcriptional regulator negatively regulated by TGF-β signaling [28]. Loss of DAF-3 by genetic null does not result in locomotion or embryonic lethality phenotypes, but rather, dauer defective animals [31]. The other predicted targets of PAT-9/ZTF-19 are unlikely candidates for producing the Pat phenotype; *cog-1* is expressed in neurons [32], *let-7* is a heterochronic switch gene only expressed in L3, L4 and adults [33], and the *mir-76* microRNA is expressed in early embryos and adult neurons but not body wall muscle lineages [34]. This expression information along with the ChIP data has enabled us to hypothesize that PAT-9 is involved in the regulation of both *tbx-2* and *daf-3* expression, however, neither gene is responsible for the Pat phenotype of *pat-9* mutants, leaving the critical downstream target(s) of PAT-9 yet to be identified.

PAT-9 is required for proper organization of myofilaments and for recruitment to the M line. In *pat-9* mutants, UNC-52/perlecan, the most upstream molecule of the muscle attachment assembly pathway, is present in the basal lamina; however, PAT-3/integrin is not well organized at the basal membrane. Subsequently, PAT-4/ILK and DEB-1/vinculin are not recruited properly to the nascent attachment sites. The correct assembly of dense bodies and M lines are dependant upon the recruitment of each protein component in a distinct order, such that failure of one protein to assemble hinders the recruitment of all other proteins downstream in the pathway [16,17]. Therefore it is reasonable to make the prediction that the molecular components at muscle attachment sites downstream of integrin, including UNC112, UNC-97/PINCH, and PAT-6/actopaxin, would not be assembled properly at the attachment sites of *pat-9* mutants. In both *pat-4* and *pat-6* mutants, the PAT-3/integrin is not affected, since both proteins are downstream of PAT-3 in the assembly pathway. However, in *pat-9* mutants integrin is not properly organized at the basal membrane, which directly causes the disruption of the integrity of the attachment complex and expression of UNC-52 is not enough to initiate the assembly of muscle at attachment sites. Since integrins have two-way signaling, the lack of PAT-9 causes the loss of a critical inside out signaling mechanism, compounding the Pat-9 phenotype.

## Conclusions

The assembly of proteins forming muscle attachments is a highly ordered process, but is also regulated at the transcriptional level by transcription factors. Our study shows that PAT-9/ZTF-19 is indispensable for establishing and maintaining the integrity of muscle attachment sites and most likely functions at the level of transcription regulation.

## Methods

### Strains and genetics

Standard methods were used for culturing *C. elegans*[35]. The following strains were used: wild-type worms were N2 strain of the Bristol variety; RW1385 (*mnDp1(X; V)/+V*; *unc-3*(*e151*) *pat-9*(*st558*) *X*). SNP mapping was performed as described [36]. RW1385 worms were mated with Hawaiian CB4856 males and Unc non-Pat recombinants were isolated and used for PCR and RFLP analysis around known SNPs.

Because homozygote *pat-9* is lethal, a segment of duplication of the X chromosome (*mnDp1*) containing wild-type *pat-9* and *unc-3* genes was fused to one of its V chromosomes. This strain segregates 25% pats, 25% early arrested embryos containing two duplications and 50% normal progeny with the same genotype as RW1385. For the *pat-9* rescue line (+/+*V*; *unc-3*(e151) *pat-9*(*st558*) *X*; *pat-9::gfp*), the rescue animal lost the duplication *mnDp1* and is homozygous for *unc-3* and *pat-9*. The Pat phenotype was rescued by a wild-type *pat-9* gene in an extrachromosomal array and was homozygous for *unc-3*, yielding an Unc phenotype and coiled tail. The extrachromosomal arrays contain the dominant transformation marker pRF4(*rol-6*); therefore the rescued strain segregates either rollers with coiled tail or Pats.

### Molecular biology

All PCR fragments used to generate expression plasmids were first subcloned into pGEM-T easy (Promega) and sequenced. All oligonucleotide primers are listed in Additional file 1: Table S3. Cosmid T27b1 was verified to contain the whole T27b1.2 gene by sequencing with primers BW-580, BW-581, BW-582 and BW-583. For the initial transformation rescue experiment, the *pat-9* 3.3 kb promoter and coding region were amplified separately with primer pairs (BW-608, BW-610), and (BW-611, BW-612) respectively, using cosmid T27b1 as the template. Two PCR fragments have a 500 bp overlap. The initial *pat-9* full-length cDNA was generated by RT-PCR using Superscript III/Platinum Taq one-step RT-PCR system (Invitrogen) and primer pair (BW-631, BW-632). Total RNA was isolated from N2 worms using a TRIZOL (Invitrogen) based method with slight modification: 800ul Trizol was added to 200ul packed worms, vortexed, and incubated at room temperature for 20 min. The reaction was centrifuged at full speed at 4°C for 10 min, the supernatant containing the RNA was removed to a new tube, purified as per manufacturers instructions, and RNA was suspended in 10 μl DEPC-H_2_O.

To study the expression and localization of PAT-9, a *pat-9::gfp* fusion was constructed as follows. First, the 3.3 kb *pat-9* promoter was PCR-amplified using primers BW-613 and BW-630. This fragment was digested with PstI and KpnI, and then replaced the *myo-3* promoter of pPD118.20 (Addgene, Fire Lab *C. elegans* Vector Kit), to make *P*_*pat-9*_*::gfp*. The *pat-9* genomic fragment including 980 bp downstream of the 6^th^ exon was PCR-amplified using primers BW631 and BW596, digested with KpnI and DraIII, and then replaced the KpnI – DraIII region of *P*_*pat-9*_*::gfp*, to make *P*_*pat-9*_*::pat-9*. Finally, *gfp* was amplified and digested with KpnI, and inserted into *P*_*pat-9*_*::pat-9*, to make *P*_*pat-9*_*::pat-9::gfp*. The *pat-9* promoter and genomic coding region were amplified using cosmid T27b1 as a template; *gfp* was amplified using vector pPD118.20 as a template.

The following transgene plasmid constructs for NLS identification were made in the pPD118.20 vector with *gfp* fused in-frame to the carboxyl terminus of the *pat-9* coding sequence. Deletions of *pat-9* were amplified from the *pat-9* cDNA and cloned in-frame with *gfp* by NotI and KpnI digestion. The following primer pairs were used for PCR amplification: pat-9 C (primer 2 and primer 3), pat-9CNLS (primer), pat-9zf1(PJ-101, PJ-105), pat-9zf123NLS (primer 1 and primer 4). The above PCR fragments were digested with NotI and KpnI and inserted into the vector pPD118.20 NotI and KpnI site to make *P*_*myo-3*_*::pat-9 C::gfp*, *P*_*myo-3*_*::zf1::gfp* and *P*_*myo-3*_*::zf123::gfp* respectively. All plasmids were verified by sequencing. Transgenic lines were made using the standard microinjection approach with pRF4(rol-6) as an injection marker.

### PAT-9 antibody

A partial *pat-9* cDNA fragment (carboxyl terminal, amino acids 176–470) was PCR amplified (using primers PJ-117 and PJ-118) from the *pat-9* cDNA, digested with NdeI and NotI, sub-cloned into a NdeI and NotI digested pET23a plasmid (Novagen), and transformed into *E. coli* BL21(DE3) bacteria for expression. Recombinant protein was purified under denaturing conditions using Talon Resin (Clontech) as per manufacturer’s instructions, dialyzed with PBS, and used as an immunogen. Rabbit polyclonal antibodies were generated at the Immune Resource Center at the University of Illinois at Urbana-Champaign. Antiserum was affinity purified against recombinant PAT-9 C-terminal protein covalently linked to cyanogen bromide-activated sepharose (GE Healthcare) and analyzed by immunoblotting for specificity (Additional file 1: Figure S2).

### RNA interference

RNAi for all of the nine candidate *pat-9* genes were performed as previously described by dsRNA injection [17,37] and additionally for T27b1.2 by feeding bacteria induced to express double-stranded RNA to the worms [38]. The cDNA clones *yk64f5yk414e5yk443f12yk483d11yk668g10yk782b11*, and *yk839g10* were kindly provided by Dr. Yuji Kohara, National Institute of Genetics, Mishima, Shizuoka, Japan. RT-PCR from N2 RNA was performed to obtain the cDNAs for T25D1.2 (BW-570, BW-571) and F59C12.3 (BW-572, BW-573). For injection RNAi, sense and antisense RNA were generated by in vitro transcription using T3 and T7 RNA polymerase (Promega). The RNAs were mixed (1 μg/μl), injected into N2 hermaphrodites, and eggs laid between 12 – 48 h after injection were scored for the Pat phenotype. For feeding RNAi, the T27b1.2 cDNA was cloned between the two T7 promoters of vector pL4440 (Open Biosystems). The plasmid was transformed into competent HT115(DE3) bacteria, grown up from single colonies in 2x YT media containing tetracycline and kanamycin, and expression of dsRNA was induced with 1 mM IPTG when the culture reached OD600 = 0.3 ~ 0.4. NGM plates, made fresh with 50 μg/ml kanamycin, 12.5 μg/μl tetracycline and 0.4 mM IPTG, were seeded with the induced cultures and set at room temperature overnight. L4-stage hermaphrodite worms were placed on the plates and incubated for 48 h at 18°C after which 5 worms were transferred separately onto plates seeded with the same bacteria and allowed to lay eggs for 24 h at 18°C before being removed. Progeny from both the first plates and second plates were scored for a Pat phenotype and any other developmental abnormalities after another 24 h.

### ChIP

The ChIP experiments were performed essentially as described [39]. N2 worms were grown in 500 ml S medium at 20°C with shaking—producing mixed stage cultures, collected, washed, and fixed with formaldehyde. Approximately 0.4 ml of packed N2 worms were used for each ChIP experiment and each ChIP replicate experiment started with a new liquid culture. After fixation, soluble chromatin was generated by sonication, resulting in an average of 750–500 bp DNA fragments as determined by agarose gel. For the immunoprecipitation step, 10% of the soluble chromatin was removed and used as input control and the remaining chromatin was divided into two equal pools. The PAT-9 was added (1:100) to one tube and NRS (1:100) was added to the second sample as a control for non-specific binding. IPs proceeded overnight at 4°C with end-over-end rotation. The immune/chromatin complexes were collected using pre-locked protein A/G agarose (Santa Cruz Biotech). After washing extensively, the bound DNA was purified and subjected to qPCR using SYBR Green and a BioRad I-Cycler. PCR primers were designed to the 5′ regulatory regions of *frg-1* (PJ-111, PJ-112), *daf-3* (PJ-113, PJ-114), and *tbx-2* (PJ-115, PJ-116), and tested for conditions resulting in a single PCR product of the expected size.

### Embryo immunostaining

Populations of embryos were fixed and stained as previously described [40]. The following monoclonal antibodies were obtained from the Developmental Studies Hybridoma Bank and diluted as indicated: MH2 (1:100), MH25 (1:250), MH24 (1:200), and MH27 (1:1500) (developed by R. H. Waterston), and 5–6 (1:200) (developed by H. F. Epstein). The mouse polyclonal PAT-4 antibody [16] and rabbit polyclonal PAT-9 antibody were generated in the lab as described. Antibodies were diluted for staining in PBS supplemented with 0.5% tween-20 and 30% normal goat serum. Affinity-purified goat anti-mouse IgG conjugated to rhodamine, diluted 1:100 (Chemicon International) or AlexaFluor594 goat anti-rabbit IgG, diluted 1:800 (Invitrogen Corp) was used as a secondary antibody.

### Adult worm immunostaining

Immunostaining on adult animals was essentially as described [41]. Mixed stage N2 worms were harvested and washed thoroughly in PBS to remove bacteria. Worms were suspended in 4% paraformaldehyde in 100 mM sodium phosphate buffer and quick frozen on dry ice. After thawing on ice, animals were incubated on ice for 1 h, washed three times in 1% triton X-100, 100 mM tris (pH 7.5), then incubated in 1% triton X-100, 100 mM tris (pH 7.5) and 1% β-mercaptoethanol at 37°C for 2 h. Worms were washed three times in 10 mM NaBO_3_ (pH 9.2), incubated for 1 h in 10 mM NaBO_3_ + 0.3% H_2_O_2_, washed 3 times with 10 mM NaBO_3_ (pH 9.2) and stored for further processing in AbA buffer (1 × PBS, 0.1% triton X-100, 1% BSA, 0.05% NaN_3_). The PAT-9 antibody was diluted 1:500 in AbA buffer, incubated overnight at 4°C, washed three times with AbB buffer (1 × PBS, 0.1% triton X-100, 0.1% BSA, 0.05% NaN_3_) and incubated with secondary antibody (Alexa 488 goat anti-rabbit) at 4°C overnight. After washing with AbB three times, the pellets were mounted on slides for fluorescence microscopy.

## Competing interests

The authors declare that they have no competing interests.

## Authors’ contributions

BDW designed the overall study and performed the original Pat screen that isolated *pat-9*. All authors performed experiments. QL, TIJ, RAB and PLJ drafted the manuscript. All authors read and approved the final manuscript.

## Supplementary Material

Additional file 1 Characterization of the affinity purified PAT-9 rabbit polyclonal antibody. Western blots of total protein extract from A) *pat-9* or N2 embryos, or B) pat9::gfp transgenic embryos probed with the PAT-9 polyclonal antibody or GFP antibody as indicated. Arrowhead indicates PAT-9 and arrow indicates PAT-9::GFP fusion protein. Table S1. SNP Mapping results. Table S2. Nine candidate *pat-9* genes based on SAGE data. Table S3. Oligonucleotide primers. Click here for file
