# Supplementary material for: C. elegans PAT-9 is a nuclear zinc finger protein critical for the assembly of muscle attachments
Source: Cell Biosci. 2012 May 22;2:18. doi: 10.1186/2045-3701-2-18 (PMC3419604; doi:10.1186/2045-3701-2-18)
Supplement: Additional file 1 — Characterization of the affinity purified PAT-9 rabbit polyclonal antibody. Western blots of total protein extract from A) pat-9 or N2 embryos, or B) pat9::gfp transgenic embryos probed with the PAT-9 polyclonal antibody or GFP antibody as indicated. Arrowhead indicates PAT-9 and arrow indicates PAT-9::GFP fusion protein. Table S1. SNP Mapping results. Table S2. Nine candidate pat-9 genes based on SAGE data. Table S3. Oligonucleotide primers. [file 2045-3701-2-18-S1.pdf]

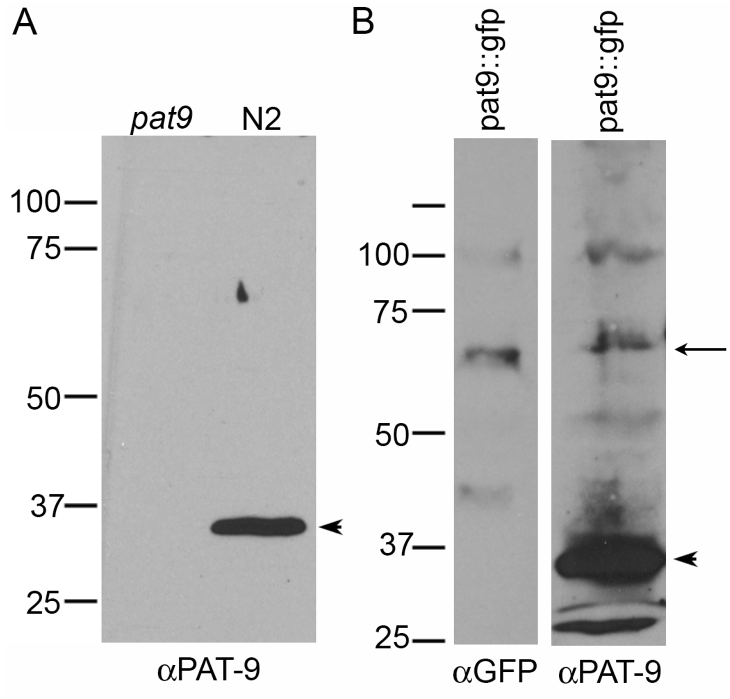

Figure S1. Characterization of the affinity purified PAT-9 rabbit polyclonal antibody. Western blots of total protein extract from A) *pat-9* or N2 embryos, or B) pat9::gfp transgenic embryos probed with the PAT-9 polyclonal antibody or GFP antibody as indicated. Arrowhead indicates PAT-9 and arrow indicates PAT-9::GFP fusion protein.

Supplemental Tables

Table S1. SNP Mapping results

SNP Marker	Recombinants N2 sequence	Recombinants HA sequence	Recombinants Total
F09B12	142	47	189
W09B12	5	184	189
T24D11	6	164	170
F38E9	5	165	170
T25D1	0	170	170
F35B3	0	189	189

Table S2. Nine candidate *pat-9* genes based on SAGE data

Candidate Gene	Size (bp)	Protein	cDNA
F38E9.5	1002/5132	WP:CE28307	yk483d11.3
F38E9.2 (nas-39)	2856/14508	WP:CE30977	yk414e5.3
F38E9.1	1710/3841	WP:CE28305	yk839g10.3
K05G3.3 (cah-3)	741/1872	WP:CE04755	yk443f12.3
T25D1.2	1317/2154	WP:CE05018	RT-PCR from N2
T27B1.2 (ztf-19)	1848/3962	WP:CE05028	yk64f5.5
F59C12.1 (cdh-9)	1797/5187	WP:CE04682	RT-PCR from N2
F59C12.3	1641/6141	WP:CE35728	yk782b11.5
C06G1.4 (ain-1)	1926/3692	WP:CE03967	yk668g10.5

Table S3. Oligonucleotide primers

Primer	Sequence
BW-570	5'-ATGCCTCGTACCAGCTTCAGAGAC
BW-571	5'-TCAGATATCACCTTTTCTAATGAATG
BW-572	5'-ATGTATCAGGGAGAGACGAACATT
BW-573	5'-TCAAGAAAATTGGTTATCCGCAAG
BW-580	5'-GGTACTGAACCTGACTGAACTTGATTGAACCTGACTGAAAAT CCTCCCTC
BW-581	5'-CATAATCAATATCAATTCAAAATTATCGATTTTATCGAAAAG TTGTCGCT
BW-582	5'-ACTTCCCGACTCTATACTCACCTCACAAATGATGAGGTGCCC AGCCAATT
BW-583	5'-GGGGGGGACGTCAAATAGTCCGGCGTTAAAGGCTCATGGGA TTAACTTTCGG
BW-596	5'-AAAAAACACATTGTGAGCTGCCGTTCGCATTCCGTACATTTA TGT TAG TTG C
BW-608	5'-GGGGGGGGCGCCGCATCTAAAATCACTCATCAAAAATAAG
BW-610	5'-GTTATTGTTCATTGGGTCCGTGTGGGTC
BW-611	5'-CATCATCCTCCCTTCTTTCCAACTTTGTCA
BW-612	5'-TTAACTTTCGGTAATTGCCAAACGCACTTT
BW-613	5'-GGGGGGCTGCAGTTAAGTTGTTGTGTTTTGTTTTGTT
BW-625	5'-CCCCCCGGTACCATGAGTAAAGGAGAAGAACTTTTCACTGG AGTTGTCCCAATTCT
BW-626	5'-AAAAAAGGTACCCGCCGCCGCCGCTGGGCTTTTGTATAGTT CGTCCATGCCATGTGTAATCCC
BW-630	5'-AAAAAAGGTACCGGTGACAAAGTTGGAAAGAAGGGAGGAT GATGGAAC CCGTGA
BW-631	5'-AAAAAAGGTACCATGGAGAATCGGACGCCGATGCAACACC ACAGTGGGTA
BW-632	5'-CTACACAGAGAGATCACTGC
PJ-101	5'-GCGGCCGCATGGAGAATCGGACGCCGAT
PJ-102	5'-GCGGCCGCACATATGCCACAGCAGCGAA
PJ-103	5'-GGTACCCACTGCTCATGTGGCACAGT
PJ-104	5'-GGTACCAATGTAGTTGCCAAGGAGCT
PJ-105	5'-GGTACCGGCTTCTGGCCGGTGTGAGA
PJ-106	5'-ATGGAGAATCGGACGCCGAT
PJ-107	5'-ATGAACTCTTGATCAGTGTT
PJ-108	5'-GGCAGCGAGAGCTCGACCAT
PJ-109	5'-CTTGTGATTCCAGAGTGTCG
PJ-110	5'-TGTCGTCGTGATCAGTGGAT
PJ-111	5'-CGGCGAGAGCGAAACATTGG
PJ-112	5'-TTCCCGTAAATCCTGCAGCTCTC
PJ-113	5'-CCGAGGTCATGTGCACTCTCTT
PJ-114	5'-TCTTCGCCCAGCACACTATT
PJ-115	5'-GACCCATTAAAAAACCACGCGCC

PJ-116	5’-ATGGTCCATGGATGGATGCCAC
PJ-117	5’-CATATGAATGGAGGACTTCCAGCATCATCG
PJ-118	5’-GCGGCCGCCCACTGCTCATGTGGCACAGTCTT
